# Circ-ZDHHC5 Accelerates Esophageal Squamous Cell Carcinoma Progression *in vitro* via miR-217/ZEB1 Axis

**DOI:** 10.3389/fcell.2020.570305

**Published:** 2020-12-17

**Authors:** Qian Wang, Lili Yang, Yanxin Fan, Weiwei Tang, Handong Sun, Zhipeng Xu, Jian Zhou, Yanzhou Zhang, Bin Zhu, Xiufeng Cao

**Affiliations:** ^1^Department of Oncology Surgery and Cancer Centre, Affiliated Taikang Xianlin Drum Tower Hospital, Nanjing University School of Medicine, Nanjing, China; ^2^Department of Oncology Surgery, Nanjing First Hospital, Nanjing Medical University, Nanjing, China; ^3^Research Unit Analytical Pathology, Helmholtz Zentrum M nchen, German Research Centre for Environmental Health (GmbH), Neuherberg, Germany; ^4^Department of Radiation Oncology, Nanjing Jiangsu Cancer Hospital, Jiangsu Institute of Cancer Research, The Affiliated Cancer Hospital of Nanjing Medical University, Nanjing, China

**Keywords:** ESCC, circ-ZDHHC5, miR-217, ZEB1, sponge

## Abstract

Circular RNA (circRNA) exhibits a covalently closed circular conformation and is structurally stable. Nevertheless, the precise effects exerted by circRNA in esophageal squamous cell carcinoma (ESCC) remains uncertain. circRNA was ascertained by a human circRNA array study and was confirmed by the quantification of reverse transcriptase polymerase reactions. A luciferase reporter, fluorescence *in situ* hybridization experiment was exploited to explore the interaction between circ-ZDHHC5 and miR-217. The function of circ-ZDHHC5 was determined by siRNA-mediated knockout of circ-ZDHHC5 in *in vitro* proliferation, migration, and invasion. circ-ZDHHC5, rather than linear ZDHHC5 mRNA, rose in the tissues of patients with ESCC, plasma, and ESCC cell lines in comparison with normal controls. Knockdown of circ-ZDHHC5 inhibited tumorigenesis in ESCC cells, and the co-transfection of si-circ-ZDHHC5 and miR-217 mimics further enhanced the above effect. Noticeably, the present study showed that circ-ZDHHC5 was an miR-217 sponge that modulated the expression of zinc finger E-box binding homeobox 1 (ZEB1), further facilitating ESCC tumorigenesis. As revealed by this study, circ-ZDHHC5 can act as a new potential circular biomarker for detecting ESCC. It provides a novel perceptivity for the treatment of ESCC suggesting that circ-ZDHHC5 could impact on ESCC progression by sponging miR-217 with ZEB1.

## Introduction

Esophageal carcinoma (ESCA) refers to a malignant tumor whose incidence is ranked 9th in a wide range of cancers (Miller et al., 2016). There are over 400,000 cases of esophageal cancer deaths each year, which ranks this type of cancer 6th in overall malignant tumor deaths (Torre et al., 2015). In China, esophageal squamous cell carcinoma (ESCC) is the primary pathology-related form of esophageal cancer, while in western countries, esophageal adenocarcinoma is more common. Over the past few years, though the therapeutic effect of ESCC has been enhanced, as fueled by the development of multidisciplinary comprehensive treatment, the long-term survival of ESCC patients continues to be low. One- and five-year cause-specific survival rates for ESCC are 43.8 and 18.9%, respectively, with a median survival of 10 months (Then et al., 2020). For ESCC patients with fewer early symptoms, diagnosis was confirmed in the advanced stage of the disease and led to lymphatic metastasis. The lack of early diagnosis of molecular markers in tumors, as well as the extreme likelihood of metastasis, are the main factors contributing to the failure of ESCC treatment. Accordingly, it is of high clinical importance to develop new early diagnostic molecular markers involved in the regulating, proliferating, and invading process of ESCC cells, and to further study their functions and mechanisms.

circRNAs are capable of forming unique continuous covalent closed loops, i.e., they exhibit no 5′ to 3′ polarity, let alone a polyadenylated tail, which is different from linear RNAs (Chen and Yang, 2015). circRNA is steady, preserved, and considerable in a variety of cell lines or cancer tissues (Jin et al., 2016; Lu and Xu, 2016). The molecular mechanism studies on circRNAs have shown that circRNAs are able to modulate downstream genes related to disease through binding with miRNAs as competitive endogenous RNA (ceRNA) (Wang et al., 2017; Zheng et al., 2017). In accordance with Shi Y and colleagues, hsa_circ_0006168 is likely to control the Mammalian Target expression of Rapamycin (mTOR) by sponging miR-100 in ESCC (Shi Y. et al., 2019). Shi N. et al. (2019) revealed that the circ-PRKCI/miR-3680-3p/AKT3 regulating system was critical to ESCC, which shed light on ESCC pathogenesis.

Based on the results of the circRNA array on ESCC plasma samples, this study first identified a circular RNA formed from the ZDHHC5 gene, which are termed as circ-ZDHHC5. In comparison with the normal control groups, circ-ZDHHC5 was subsequently reported to have a higher expression in the tissues and plasma of ESCC patients as well as in the ESCC cell lines. The experiments *in vitro* demonstrated that circ-ZDHHC5 can act as the miR-217 sponge to modulate zinc finger E-box binding homeobox 1 (ZEB1) expression, thereby promoting ESCC cells tumorigenesis. The present study issues a perspicacity of the ESCC treatment and suggests a probable circulation biological marker to identify ESCC at the early stage.

## Materials and Methods

### Patient Samples

The 24 samples of ESCC tissues, which were overall consistent with adjacent general tissues, and the 20 samples of 10 ml venous blood before the operation were harvested from the ESCC cases at the General Surgery Department of Nanjing Hospital affiliated to Nanjing Medical University from 2015 to 2018 abiding by the Helsinki Declaration. A total of 15 specimens of 10 ml normal venous blood were collected in a random manner from the individuals aged 50–90 years with no potential disease at the Physical Examination Center of Nanjing Hospital from 2015 to 2017. All the mentioned samples were frozen in liquid nitrogen and steadily maintained at −80°C until the RNA was extracted. Informed consent of the mentioned patients was acquired before the samples were collected. This project received approval from the Ethics Committee of Nanjing Medical University.

### Quantitative Reverse Transcription Polymerase Reaction (qRT-PCR)

In accordance with the directive of the producer, overall RNAs from cells, plasma, and tissues were isolated with a Trizol Reagent (Invitrogen, United States). In terms of mRNA and circRNA, cDNA was generated with a reverse transcription kit (Vazyme, China) and for miRNA, overall RNAs showed reversion with a RiboBio reverse transcription kit (Guangzhou, China). mRNA, circular RNA, and miRNA PCR were quantified with a SYBR Green PCR Kit (Takara, Japan). All primer sequences were designed and generated by Tsingke (Beijing, China). circ-ZDHHC5 expression was explored with the primers below: 5′- TTCCTTGATCCCTGGACCAG -3′ (Forward) and 5′- CCCCACGACTCAACTGGTAA -3′ (Reverse). The primers of ZDHHC5 are 5′- CACCTGCCGCTTTTACCGT -3′ (Forward) and 5′- CGGCGACCAATACAGTTATTCAC -3′ (Forward). GAPDH was employed for normalizing circRNA and mRNA expressions before calculation.

### RNase R and Actinomycin D Treatment

For the RNase R treatment process, 2 mg of overall RNA underwent incubation for 20 min at 37 in the presence or absence of 3 mg of RNase R (Epicenter Technologies, Madison, WI) with the RNeasy MinElute cleaning Kit (Qiagen, China). The resulting RNA then underwent purification. In the actinomycin D treatment, 2 mg of overall RNA underwent incubation at 37 in the presence or absence of 1 mg actinomycin D (Sigma, Chengdu best reagent company), and the acquired RNA was determined at 6, 12, and 18 h, respectively.

### Cell Culture and Transfection

The ESCC cell lines primarily originated from the Shanghai Institutes for Biological Sciences, Chinese Academy of Sciences. A human normal esophageal cell line termed as HET-1A was donated by Dr. Zhihua Liu at the State Key Laboratory of Molecular Oncology, Cancer Institute, Chinese Academy of Medical Sciences (Beijing, China). HET-1A is the cell line originally derived from the normal esophageal epithelium of a 25-year-old male. TE-10 and TE-12, the main cell lines used in this study, are both human esophageal cancer cells with epithelial-like and adherent growth characteristics. A 50 nM siRNA used as a negative control (NC) and circ-ZDHHC5 (si-circ-ZDHHC5) by the Lipofectamine 2000 transfection reagent (Invitrogen, United States) were employed in transected TE-10 and TE-12 cell lines complying with the directive of the producer. All the cells in this study underwent the culturing process in RPMI 1640 medium (GIBCO BRL, United States) with 10% fetal bovine serum (FBS) at 37°C in a moisturized incubator supplemented by 5% CO_2_. The sequences of circ-ZDHHC5 siRNAs (RIBOBIO, China) included: siRNA-1: siRNA-3: 5′- TTACCAGTTGAGTCGTGGG -3′; siRNA-2: 5′- TGCGTTACCAGTTGAGTCG -3′; 5′- CTTCTGCGTTACCAGTTGA -3′. Ultimately the knockdown efficiency of circ-ZDHHC5 was ascertained with qRT-PCR.

### Nucleus-Cytoplasm Fractionation

Firstly, 1 × 10^6^ ESCC cells were cleaned two times with pre-cooled PBS. Subsequently, the cell layer underwent the scrapping process in 500 μl PBS and centrifugation at 500 *g* for 5 min at 4°C. Subsequently, the supernatant removal was achieved, and the cleaning procedure was carried out two times. Lastly, nuclear and cytoplasmic RNA from cultured ESCC cells underwent the isolation by PARIS KIT50 RXNS (life, AM1921) in accordance with the directives of the producer. The relevant mRNA was, respectively, identified as a control of cytoplasmic RNA and nuclear RNA in the isolated RNA. Biotriples were employed, and subsequently qRT-PCR was carried out for assessing the corresponding mRNA and circRNA levels.

### Luciferase Reporter Experiment

The wild-type and mutant fragments related to the miR-217 binding site in circ-ZDHHC5 3′-UTR were designed and generated, and then inserted into the pGL3-basic vector (Realgene, China). 293T cells were seeded into cell culture plates. When the cell density reached nearly 70%, pGL3-basic vector and miR-217 mimics or inhibitors were transfected. Three replicates were set for the respective sample. At 36–48 h after transfection, the culture medium was discarded, and the reagent was added to lyse the cells. Lastly, luciferase experiment reagent II was added and the luciferase activity in the samples were identified and analyzed.

### Fluorescence *in situ* Hybridization (FISH)

FISH was conducted for detecting circZDHHC5 presence with a Cy3-labeled probe (5′CY3-TTCAAGCTGTCCCCAC GACTCAACTGGTAACGCAGAAGAGG3′) and miR-217 with a FITC-labeled probe (5′FITC-TCCTCGTTCCTGTGCGT3′), separately. After prehybridization, samples underwent the hybridization in the buffer with specific probes at 37–40 in an incubator for 2–4 h. Next, 4,6-diamidino-2-phenylindole (DAPI) was introduced for 5 min to label the nuclei. Lastly, the slides were mounted with a fluorescent protective reagent and the images were acquired as soon as possible.

### Clone Formation Experiment, Cell Counting Kit-8 (CCK-8) Proliferation Experiment, and 5-Ethynyl-20-Deoxyuridine (EdU) Incorporation Experiment

In the clone formation assay, transfected cells underwent the seeding process in 6-well plates at a density of 1,000 cells per well and the culturing process in RPMI 1640 medium supplemented by 10% FBS. Ten days later, the cells were fixed with methanol and then stained with GIEMSA. Lastly, the images of colonies were made, and the counting process of colonies was carried out. For the CCK-8 experiment, ESCC cells were seeded in 96-well plates at 4,000 cells per well. Seeded cells were treated with 10 μl of CCK-8 solution (EnoGene, China) and cultured for 0, 24, 48, 72, 96 h, respectively. Subsequently, the cell absorbance was analyzed at 450 nM by a microplate reader (BioTek, United States) following the producer’s instructions. For the identification of cell proliferation with EdU, logarithmic growth cells were inoculated into 96-well plates, polylysine was added after being cultured to a normal growth stage and incubated at 37°C in an incubator for 4 to 7 h. The polylysine was discarded, and the cells were permeabilized and fixed with a penetrant (PBS supplemented by 0.5% TritonX-100), a glycine solution, and a cell fixing solution (PBS supplemented by 4% paraformaldehyde) after drying. The cells were transfected in accordance with the needs of the experiment, EdU-labeled and fixed after 24–48 h, and lastly stained. The images were explored by laser confocal microscopy.

### Transwell Invasion Experiments

A total of 10 μL of fibronectin (0.5 mg/mL) was applied to the bottom of respective Transwell cells and air-dried. A total of 50 μL of matrix gel was added to the respective well. Then, 10^5^ transfected cells were placed in 1.5 mL EP tubes and centrifuged at 2,000 rpm for 5 min. The supernatant was eliminated. Afterward, 200 μL of serum-free 1640 medium was added to resuspend the cells, and then added to the Transwell chamber. The lower chamber was filled with 1640 medium with 20% serum, and then incubated at 37°C in an incubator for 24 h. The Transwell chamber was eliminated, the cells inside were wiped with a cotton swab, and the remaining cells were gently washed away with PBS. A mixture of 3: 1 was prepared with methanol and glacial acetic acid, and the cells were fixed on the opposite side of the Transwell chamber for 30 min. The samples were placed in a crystal violet staining solution and stained for 15 min. The film was cleaned and fixed on a glass slide for significant observation. Three random fields were taken under the microscope to take pictures.

### Wound Healing Experiment

The TE-10 and TE-12 cells underwent a culturing process in six-well plates and a scraping process with the fine end of 200 μl pipette tips (at 0 h). At 0, 24, and 48 h after scratching, cell migration was photographed with 10 high-power fields. The remodel was ascertained as a diminishing distance across the induced injury, normalized to the 0 h control, and presented as relative migration. We measured the areas (the scratch distance is an equivalent measurement) to calculate the closure. The formulas are: average scratch width = scratch gap area/length; the relative cell migration rate = (the scratch width at 0 h – scratch width after culture)/the scratch width at 0 h × 100%.

### Western Blot

Approximately 48 h after transfection, cells were lysed with RIPA lysis buffer (Beyotime, China) to extract protein. An appropriate amount of cell protein samples were taken, and SDS-denaturation 10% polyacrylamide gel electrophoresis was performed (SDS-PAGE) until the target proteins were well separated; subsequently, electrophoresis was stopped. The gel was transferred to a 4 transfection buffer at 350 mA for 2 h, and the proteins in the gel were transferred to a PVDF membrane to form a blot; then, they were incubated with the antibodies (EnoGene, United States), respectively. Lastly, the PVDF membrane was placed in a Western LightningTM Chemiluminescence Reagent developer for 30 s, and then developed and fixed. Then, the LabWorksTM gel imaging and study network was employed to shoot and delve into the brightness value of the respective band. The analyzing method was to calculate the ratio of the ZEB1 band brightness value of the respective sample to the GAPDH (internal reference) band brightness value.

### Statistical Analysis

Data are expressed as mean ± standard deviation (SD). Statistical studies were overall determined with the SPSS 19.0 (IBM, SPSS, and Chicago, IL, United States) statistical software and GraphPad Prism 7 with Student *t* test or one-way ANOVA. For overall outcomes, *P* < 0.05 exhibited statistical significance.

## Results

### Circ-ZDHHC5 Is Noticeably Upregulated in ESCC

**FIGURE 1:**
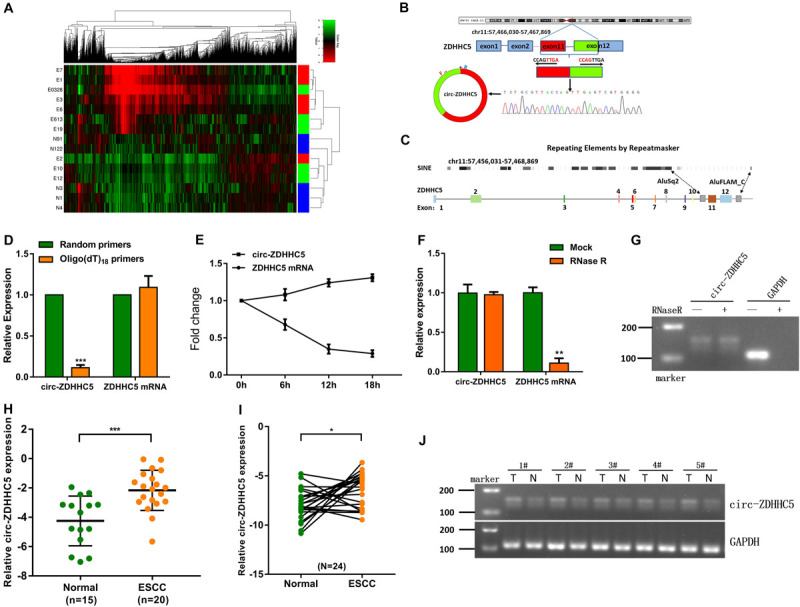
Circ-ZDHHC5 expression increases in clinical ESCC samples. **(A)** Heatmap studies on circRNA microarray data showing differentially expressed circRNA in the ESCC plasma group and normal control group. (“E” represents patient samples, and “N” represents normal control samples. “Red” represents a high level of expression, and “green” represents a low level). **(B)** The spliced mature sequence length of circ-ZDHHC5 originated from the *ZDHHC5* gene. **(C)** The genomic regions of ZDHHC5 exons 11 and 12 containing flanking Alu repeats and multiple SINEs are distributed upstream of exon 11 and downstream of exon 12. **(D)** circ-ZDHHC5 expression was noticeably downregulated with the Oligo (dT) 18 primer. **(E)** circ-ZDHHC5 had better resistance to actinomycin D than mRNA. **(F)** circ-ZDHHC5 had better resistance to RNase R than mRNA. **(G)** The agarose gel electrophoresis of circ-ZDHHC5 in ESCC cells. **(H)** circ-ZDHHC5 expression in ESCC plasma and normal plasma. **(I)** circ-ZDHHC5 expression in ESCC tissues and adjacent non-cancerous tissues. **(J)** circ-ZDHHC5 was performed by agarose gel electrophoresis in five pairs of tissues, and were normalized to GAPDH. (*p < 0.05, ***p < 0.001).

This study harvested plasma samples from 10 ESCC cases (e.g., different TNM stages) and 5 normal cases for a circRNA microarray study to delve into the expression characteristics of circRNAs. As indicated in the result, compared with other circRNAs, the expression of circ-ZDHHC5, which has never been reported before, was noticeably upregulated in the plasma from the ESCC patients (Figure 1A). The circ-ZDHHC5 was derived from the exons 11–12 of the gene ZDHHC5 and the amplification products were inserted into a T-vector for Sanger sequencing to ascertain its full length (Figure 1B). The presence of Alu repeats and complementary sequences around exons showed relationships with circRNA formation. The genomic regions of ZDHHC5 exons 11 and 12 covered flanking Alu repeats and long introns (Figure 1C). In contrast to the random primers, when oligo(dT)18 primers were adopted, the relative expression level of circ-ZDHHC5 evidently decreased, while the relative expression level of linear ZDHHC5 was not noticeably changed, indicating that circ-ZDHHC5 did not have a poly-A tail (Figure 1D). Resistance to digestion with actinomycin D (Figure 1E) or RNase R (Figure 1F) further confirmed that this RNA species was in a circular form. Agarose gel electrophoresis was employed for the verification of circ-ZDHHC5 specificity (Figure 1G). As inspired by the huge diagnostic and therapeutic effects of circRNA in ESCC, the clinical significance of circ-ZDHHC5 was explored by identifying its expression in ESCC samples. As revealed from the outcomes, circ-ZDHHC5 had noticeably higher expression in ESCC plasma (Figures 1H,J and Supplementary Figure S1A), and tissues (Figure 1I) in comparison with normal controls. The clinical characteristics of patient samples are listed in the Supplementary Tables.

### Knockdown of Circ-ZDHHC5 Plays a Tumor Suppressing Effect in ESCC Cells *in vitro*

**FIGURE 2:**
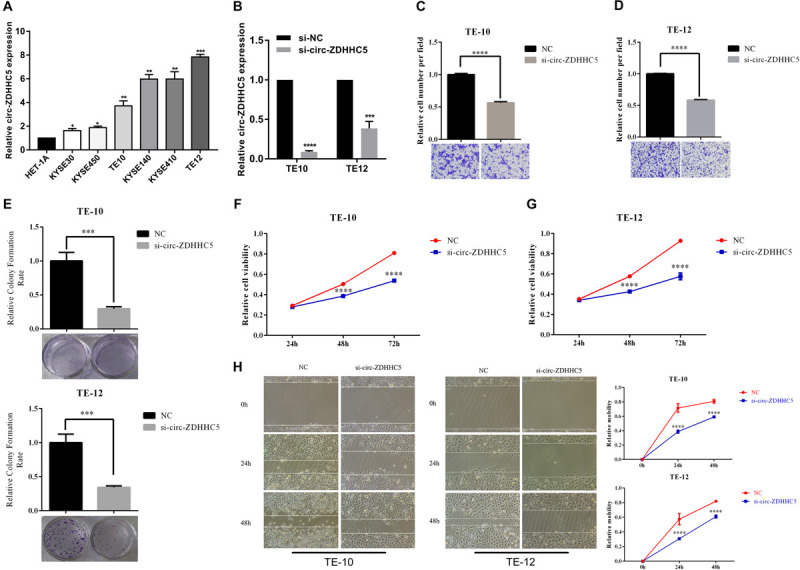
Circ-ZDHHC5 produces oncogene effects on ESCC cells. **(A)** The expression of circ-ZDHHC5 was assessed in cell lines with qRT-PCR. **(B)** circ-ZDHHC5 were knocked down effectively in ESCC cells. **(C,D)** Cell invasion experiments were conducted in cells transfected with control or si-circ-ZDHHC5. **(E)** Colony formation experiment showed the population dependence of si-circ-ZDHHC5. **(F,G)** The growth curves of cells were ascertained after transfection with si-circ-ZDHHC5 or si-NC with CCK-8 experiments. **(H)** Cell motility was determined in cells transfected with si-circ-ZDHHC5 or si-NC by the wound healing experiment. (*p < 0.05, **p < 0.01, ***p < 0.001, ****p < 0.0001).

qRT-PCR was employed to investigate the expression of circ-ZDHHC5 in ESCC cells and results showed that circ-ZDHHC5 had a noticeably higher expression in ESCC cells in comparison with normal esophageal cells HET-1A (Figure 2A). For the assessment of the role of circ-ZDHHC5 in ESCC cells, siRNA against circ-ZDHHC5 were designed to silence circ-ZDHHC5 effectively in TE-10 and TE-12 cells (Figure 2B). The result of the invasion experiment showed that the downregulation of circ-ZDHHC5 inhibited cell invasion (Figures 2C,D). The clone formation experiment and the CCK-8 experiment showed that after knockdown of circ-ZDHHC5, the proliferation ability of TE-10 and TE-12 cells was noticeably decreased (Figures 2E–G). The wound healing experiment revealed that silencing circ-ZDHHC5 noticeably decreased the cell mobility (Figure 2H).

### Circ-ZDHHC5 Directly Binds to miR-217 and Inhibits miR-217 Activity

**FIGURE 3:**
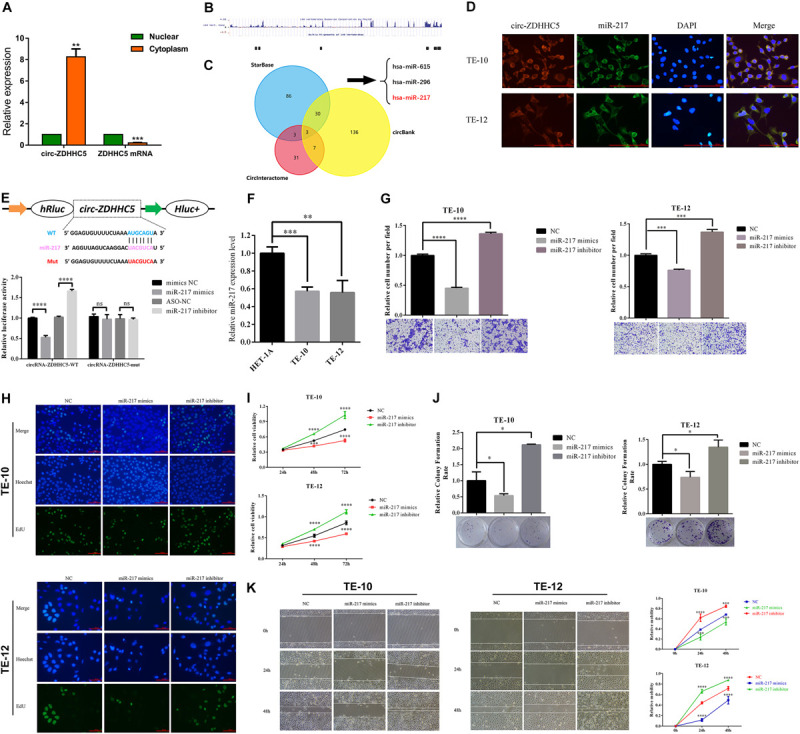
Circ-ZDHHC5 directly binds to miR-217 and inhibits miR-217 activity. **(A)** The nucleus-cytoplasm fractionation experiment showed the location of circ-ZDHHC5. **(B)** circ-ZDHHC5 was predicted to have binding sites for AGO2. **(C)** circ-ZDHHC5 might become a sponge for multiple miRNAs. **(D)** FISH showed the co-location of circ-ZDHHC5 and miR-217. **(E)** The schematic of circ-ZDHHC5 wild-type (WT) and mutant (Mut) luciferase reporter vectors. The relative luciferase activities were explored in 293T cells co-transfected with miR-217 mimics or miR-NC and luciferase reporter vectors circ-ZDHHC5-WT or circ-ZDHHC5-Mut. **(F)** The expressions of miR-217 were explored with qRT-qPCR in ESCC cells. **(G)** Cell invasion experiments were performed in cells transfected with miR-217 mimics or inhibitors with a Transwell chamber. **(H–J)** EdU, CCK-8, and colony formation experiments of ESCC cells transfected with miR-217 mimics or inhibitors were performed for the assessment of cell proliferation. **(K)** Cell motility was determined in cells transfected with miR-217 mimics or inhibitors by the wound healing experiment. (*p < 0.05, ***p < 0.001, ****p < 0.0001).

For delving into the location of circ-ZDHHC5 in ESCC cells, a nuclear and cytoplasm separation assay was carried out. It was reported that circ-ZDHHC5 was primarily located in the cytoplasm while its source gene ZDHHC5 was primarily in the nuclear (Figure 3A). The available data sets originated from doRiNA^1^; the AGO2-binding sites of the circ-ZDHHC5 genomic region were achieved, which revealed that circ-ZDHHC5 exhibited a high degree of AGO2 occupancy (Figure 3B). Given that circRNA could bind to different miRNAs and regulate downstream genes, the circBank, circinteractome, and starbase software were employed to assess possible miRNAs binding to circ-ZDHHC5 (Figure 3C). To confirm the website prediction, FISH was carried out to reveal that circ-ZDHHC5 was co-localized with miR-217 in the cytoplasm of ESCC cells (Figure 3D). It was reported that circ-ZDHHC5 exhibited a complementary sequence to the miR-217 seed region by the bioinformatics study. According to the RIP assay and dual-luciferase reporters with either the wild-type circ-ZDHHC5 sequence (WT) or the sequence with mutated binding sites of miR-217 (Mut) into the 3′ UTR of renilla luciferase, miR-217 mimics could noticeably inhibit the luciferase activities of the WT reporter while the miR-217 inhibitor played an opposite role rather than a mutant one (Figure 3E and Supplementary Figure S1C). On the whole, the mentioned evidence reveals that circ-ZDHHC5 could bind to miR-217 and inhibit miR-217 activity.

### MiR-217 Targets ZEB1 and Inhibits the Proliferation and Invasion of ESCC Cells

**FIGURE 4:**
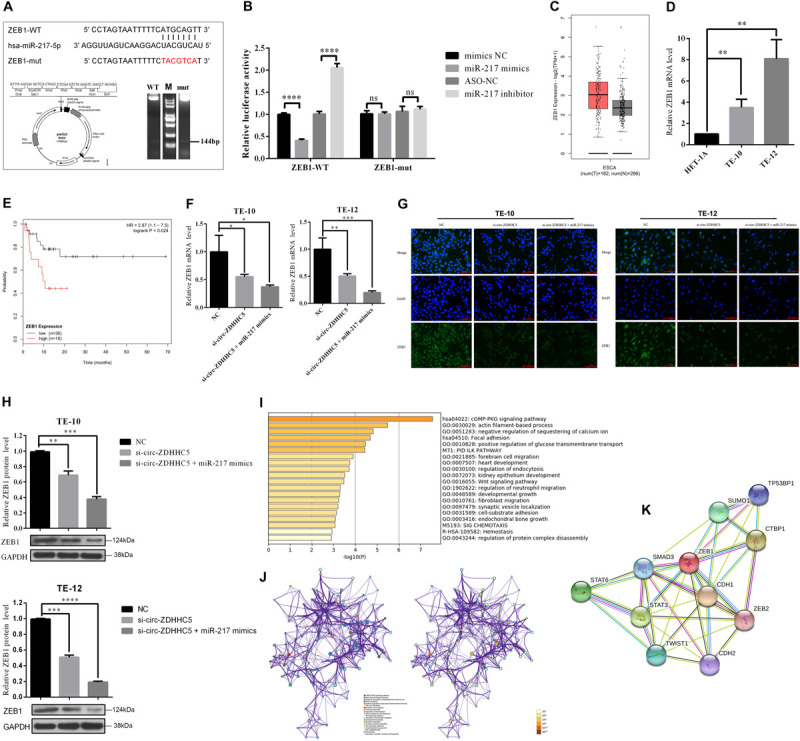
Circ-ZDHHC5 accelerates the proliferation of ESCC by sponging miR-217 for regulating ZEB1. **(A)** The schematic of ZEB1 3′ UTR wild-type (WT) and mutant (Mut) luciferase reporter vector is presented. **(B)** The relative luciferase activities were explored in 293T cells co-transfected with miR-217 mimics or inhibitors in-296-5p or in-NC. **(C)** The significant expressions of ZEB1 mRNA in ESCA tissues and normal tissue. **(D)** The relative expressions of ZEB1 mRNA with qRT-PCR in ESCC cells. **(E)** Kaplan-Meier overall survival curve of patients with more significant ZEB1 expression. **(F)** The expression levels of ZEB1 mRNA were explored with qRT-PCR in ESCC cells when co-transfected with si-circ-ZDHHC5 or si-circ-ZDHHC5 + miR-217 mimics. **(G)** The expression of ZEB1 protein noticeably decreased in ESCC cells by being co-transfected with si-circ-ZDHHC5 and miR-217 mimics. **(H)** The relative ZEB1 protein expressions were determined with Western Blot in cells transfected with si-circ-ZDHHC5 + miR-217 mimics. **(I)** Functional enrichment summary of co-expression genes with ZEB1. **(J)** Network of ZEB1 gene and the top 100 co-expression genes. The left is colored by cluster ID and nodes sharing the identical cluster are typically close to each other. The right is colored by *p*-value and terms covering more genes may achieve a more noticeable *p*-value. **(K)** The interaction network of ZEB1 and the proteins being able to bind to ZEB1 directly. (*p < 0.05, **p < 0.01, ***p < 0.001, ****p < 0.0001).

It was reported that the miR-217 expressed decreased in TE-10 and TE-12 cells (Figure 3F). The functions of miR-217 on the ESCC cells processes were studied next. As revealed from the experiments *in vitro*, miR-217 over-expression could significantly hinder the invasion (Figure 3G), proliferation (Figures 3H–J), and migration (Figure 3K) of ESCC cells, while decreased miR-217 expression might impose an opposite effect. Based on the miRanda^2^ prediction, ZEB1 mRNA 3′ UTR was reported to be targeted by miR-217 with a high score, as demonstrated by the dual-luciferase reporter experiment. According to a consequent luciferase reporter experiment, the luciferase intensity noticeably decreased after the co-transfection of the wild-type luciferase reporter and miR-217 mimics, while the mutated luciferase reporter achieved an inconsistent result. Moreover, the inhibition of miR-217 could evidently boost the luciferase activity compared with ASO-NC with the wild-type ZEB1 sequence (WT) (Figures 4A,B). The mentioned results reveal that miR-217 could target ZEB1 and partially hinder ESCC development.

### Circ-ZDHHC5 Accelerates the Proliferation of ESCC by Sponging miR-217 to Regulate ZEB1

**FIGURE 5:**
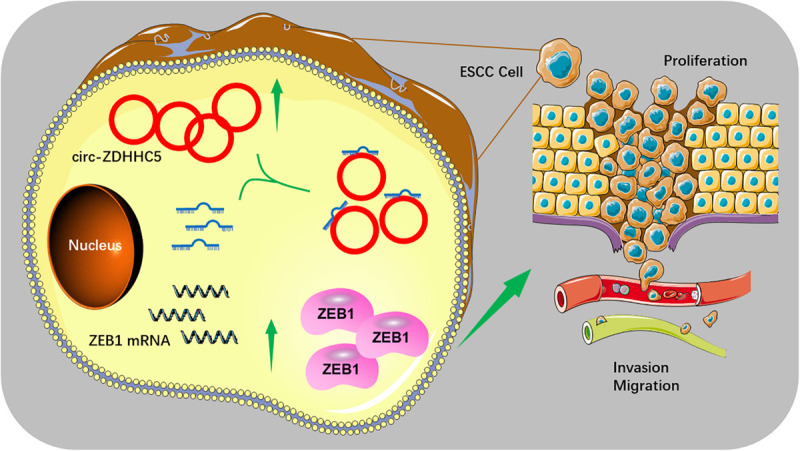
The schematic diagram of the mechanism of the circ-ZDHHC5/miR-217/ZEB1 axis in ESCC.

We initially explored the ZEB1 expression in normal tissues and ESCC tissues with TGCA. The results in TCGA and the GTEx database showed that ZEB1 has significant higher expression in ESCA tissues than that in normal tissues (Figure 4C). Moreover, we found that ZEB1 was over-expressed in TE-10 and TE-12 cells (Figure 4D). Kaplan-Meier Plotter results showed that more significant ZEB1 expression was associated with shorter OS in comparison with lower expression (Figure 4E). qRT-PCR, Western Blot, and Immunofluorescence (IF) experiments showed that the mRNA and protein expression of ZEB1 in the group treated with si-circ-ZDHHC5 + miR-217 mimics was noticeably decreased compared with the group treated withsi-circ-ZDHHC5 in ESCC cell lines (Figures F–H and Supplementary Figure S1B). Metascape was employed to perform the functional enrichment analysis of the co-expression genes of ZEB1 and the results showed that ZEB1 was primarily involved in the WNT, cGMP-PKG signal pathway (Figures 4I,J). By STRING, it was shown that ZEB1 can also directly bind to a variety of proteins (e.g., STAT3 and TWIST) regulating cell adhesion, migration and epithelial cell proliferation, and work together on cancer occurrence and progression (Figure 4K). The mentioned results demonstrate that circ-ZDHHC5 could regulate ZEB1 expression by acting as a competing endogenous RNA to sponge miR-217 and further regulate ZEB1. The mechanism of the circ-ZDHHC5/miR-217/ZEB1 is visualized in Figure 5.

## Discussion

In the present study, circ-ZDHHC5 was confirmed to have a noticeably higher expression in ESCC plasma, tissues, and cells in contrast to the normal controls. A growing amount of research has reported the relationships between circRNAs and the progression of ESCC clinically and studied the application of circRNAs as tumor biomarkers in clinical patients. For instance, Xia et al. (2016) studied a group of 51 patients and reported that hsa_circ_0067934 showed noticeable over-expression in ESCC tissues in contrast to paired adjacent normal tissues. The high hsa_circ_0067934 expression showed relationships to I-II TNM stage, I-II T stage, and poor differentiation (Xia et al., 2016). However, the majority of the studies only identified circRNAs expression in tumors and adjacent tissues. Only a few studies focused on the expression level of circRNAs of the preoperative blood from the ESCC patients, and their sample sizes were small. Our group previously reported that circ-TTC17 showed over-expression in ESCC plasmas, and the cutoff value of circ-TTC17 reached −2.548 exhibiting 88.00% specificity and 73.33% sensitivity. Moreover, circ-TTC17 in plasma was reported without any aberrant relationships to tumor size, differentiation, gender, age, or common clinical biomarkers (e.g., SCCA, CEA, and AFP) in ESCC cases. However, circ-TTC17 expression showed positive relationships to TNM stage and lymphatic metastasis (Wang et al., 2019). The outcome based on this study makes circ-ZDHHC5 an ideal non-invasive biomarker for diagnosing ESCC.

This study confirmed that circ-ZDHHC5 was highly expressed in ESCC patient samples, and that miR-217 could target ZEB1 and inhibit ESCC cells from being proliferated and invaded. Current research suggests that miR-217 is critical to cancers. For instance, He S and colleagues reported that miR-217 acted as a tumor inhibiting agent in the development of osteosarcoma (He et al., 2019). Jiang B reported that miR-217 inhibited tumor-induced M2 macrophage polarization by targeting IL-6 and regulating the JAK3/STAT3 signaling pathway, probably providing an underlying therapeutic target in the treatment of ovarian cancer (Jiang et al., 2019). In ESCC, Wang X and colleagues showed that miR-217 hindered the proliferation, migration, and invasion of ESCC cells (Wang et al., 2015). In numerous studies, circRNAs act as miRNA sponges to regulate miRNAs posttranscriptional actions to inhibit targeted mRNAs from being translated and/or stable (Yu et al., 2016; Xu et al., 2018). Li GF and colleagues suggested that the circ-DDX42/miR-761/ZIC5 axis is likely to act as a glioma treatment target (Li et al., 2018). Hao LG, and colleagues elucidated a novel circRNA (circ-DDX42) that conferred an oncogenic function in pancreatic ductal adenocarcinoma (PDAC) through the sponge of miR-625 and miR-892b (Hao et al., 2019).

We first found that knocking down circ-ZDHHC5, which suppresses the ESCC progression by modulating miR-217 and ZEB1 expression, could effectively inhibit the proliferation, invasion, and metastasis of ESCC cells. Thus far, functional inactivation of the onco-protein ZEB1 has been studied in multiple cases of ESCC, that demonstrated ZEB1 is closely linked to disease progression and prognosis. Highly expressed ZEB1 shows noticeable associations with the malignancy of various cancers (Dong et al., 2019; Shao et al., 2019; Zhao et al., 2019). Signal transduction and activation of ZEB1 is critical to cancer transformation and epithelial-mesenchymal transition (EMT) (Caramel et al., 2018). Emerging evidence reveals that ZEB1 drives EMT induction by activating stem cell traits, and evading immunity and epigenetic reprogramming. ZEB1 accelerates epigenetic silencing of E-cadherin by recruiting multiple chromatin enzymes of the E-cadherin promoter, e.g., histone deacetylase (HDAC), DNA methyltransferase (DNMT), and ubiquitin ligase (Zhang et al., 2019). Destruction of the junction between ZEB1 and the mentioned chromatin-modifying enzymes may represent an effective method of treating cancer. In the present study, Metascape was employed to perform the enrichment analysis of ZEB1 and the results showed that ZEB1 was primarily involved in the WNT, cGMP-PKG signal pathway, which indicated that circ-ZDHHC5 might promote the development of ESCC through the mentioned pathways. The mentioned findings are likely to facilitate ESCC treatment.

The present study is considered the initial work investigating the effect exerted by circ-ZDHHC5 in ESCC. However, our interpretation of the findings does have limitations. First of all, the ESCC plasma and tissue samples employed in the present study are from an identical ethnic group. Accordingly, we hope to further expand the sample size and verify the population situation in various regions. Second, this work explored the ability of circ-ZDHHC5 to bind to miR-217, whereas other miRNAs are also likely to bind circ-ZDHHC5 for regulating ESCC emergence and development. Third, whether circ-ZDHHC5 regulates the development of ESCC via other systems e.g., binding to proteins needs further exploration. We would like to clarify the diagnostic potential of circ-ZDHHC5 and its function in ESCC in subsequent projects.

## Conclusion

In brief, circ-ZDHHC5 increased noticeably in ESCC patients, and it also regulated the proliferating process, invasion, and migration of ESCC cells. It could also modulate ZEB1 expression by sponging miR-217, thereby exerting its key function in ESCC progress. The circ-ZDHHC5/miR-217/ZEB1 axis is confirmed to regulate the progress of ESCC through the ceRNA mechanism. Accordingly, circ-ZDHHC5 can act as a promising therapeutic target and prognostic biomarker in terms of ESCC.

## Data Availability Statement

The raw data supporting the conclusions of this article will be made available by the authors, without undue reservation.

## Ethics Statement

The studies involving human participants were reviewed and approved by the Ethics Committee of Nanjing First Hospital, Nanjing Medical University. The patients/participants provided their written informed consent to participate in this study.

## Author Contributions

QW and WT wrote the manuscript. QW, LY, and HS performed the experiments and analyzed data. JZ, ZX, YF, and YZ collected the plasma and tissues samples. XC and BZ reviewed the manuscript and supported the funding. All authors have read and agreed to the content.

## Conflict of Interest

The authors declare that the research was conducted in the absence of any commercial or financial relationships that could be construed as a potential conflict of interest.

## Footnotes

1 http://dorina.mdc-berlin.de

2 http://mirdb.org/
