# Supplementary material for: Circ-ZDHHC5 Accelerates Esophageal Squamous Cell Carcinoma Progression in vitro via miR-217/ZEB1 Axis
Source: Front Cell Dev Biol. 2020 Dec 17;8:570305. doi: 10.3389/fcell.2020.570305 (PMC7773775; doi:10.3389/fcell.2020.570305)
Supplement: Supplementary file 1 [file Table_1.DOCX]

**Supplementary Figure 1. (A)** The densitometry of the circ-ZDHHC5 in tissues. **(B)** The fluorescence intensity of ZEB1 protein in the treated ESCC cells. **(C)**RIP Assay was performed to detect the enrichment of circ-ZDHHC5 using Ago2 or IgG antibody. (**p < 0.05, **p < 0.01, ***p < 0.001, ****p < 0.0001*)


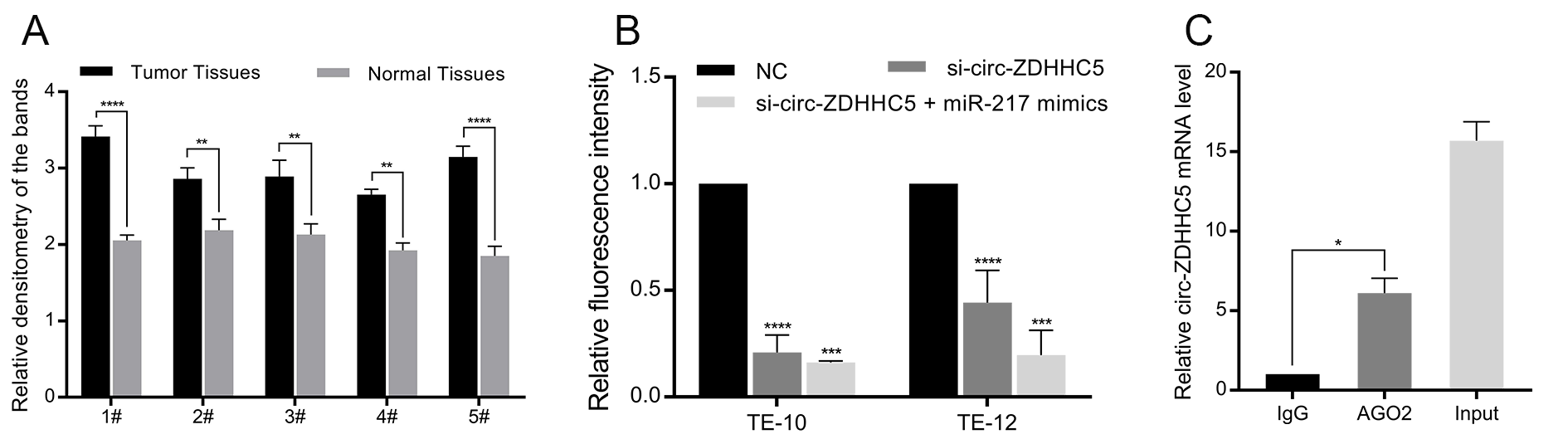


**Supplementary Tables. Clinical characteristics of patients’ samples.**

S Table 1. Clinical characteristics in 24 ESCC patients (tissues)

| **characteristic** | **case** |
| --- | --- |
|  |  |
| Age(yeas) |  |
| <65 | 14 |
| ≥65 | 10 |
| Gender |  |
| Female | 7 |
| Male | 17 |
| Tumor size(cm) |  |
| <5 | 11 |
| ≥5 | 13 |
| Histological grade |  |
| High | 9 |
| Middle-low | 15 |
| Lymph node metastasis |  |
| Negative | 4 |
| Positive | 20 |
| TNM stage |  |
| I–II | 8 |
| III–IV | 16 |

S Table 2. Clinical characteristics in 20 ESCC patients (plasmas)

| **characteristic** | **case** |
| --- | --- |
|  |  |
| Age(yeas) |  |
| <65 | 7 |
| ≥65 | 13 |
| Gender |  |
| Female | 5 |
| Male | 15 |
| Tumor size(cm) |  |
| <5 | 6 |
| ≥5 | 14 |
| Histological grade |  |
| High | 8 |
| Middle-low | 12 |
| Lymph node metastasis |  |
| Negative | 5 |
| Positive | 15 |
| TNM stage |  |
| I–II | 8 |
| III–IV | 12 |
